# An Overview on Mitochondrial-Based Therapies in Sepsis-Related Myocardial Dysfunction: Mitochondrial Transplantation as a Promising Approach

**DOI:** 10.1155/2022/3277274

**Published:** 2022-06-06

**Authors:** Behnaz Mokhtari, Rana Yavari, Reza Badalzadeh, Ata Mahmoodpoor

**Affiliations:** ^1^Drug Applied Research Center, Tabriz University of Medical Sciences, Tabriz, Iran; ^2^Molecular Medicine Research Center, Tabriz University of Medical Sciences, Tabriz, Iran; ^3^Department of Physiology, Faculty of Medicine, Tabriz University of Medical Sciences, Tabriz, Iran; ^4^Intensive Care Unit, Emam Reza Hospital, Tabriz University of Medical Sciences, Tabriz, Iran; ^5^Evidence-Based Medicine Research Center, Tabriz University of Medical Sciences, Tabriz, Iran

## Abstract

Sepsis is defined as a life-threatening organ failure due to dysregulated host response to infection. Despite current advances in our knowledge about sepsis, it is still considered as a major global health challenge. Myocardial dysfunction is a well-defined manifestation of sepsis which is related to worse outcomes in septic patients. Given that the heart is a mitochondria-rich organ and the normal function of mitochondria is essential for successful modulation of septic response, the contribution of mitochondrial damage in sepsis-related myocardial dysfunction has attracted the attention of many scientists. It is widely accepted that mitochondrial damage is involved in sepsis-related myocardial dysfunction; however, effective and potential treatment modalities in clinical setting are still lacking. Mitochondrial-based therapies are potential approaches in sepsis treatment. Although various therapeutic strategies have been used for mitochondrial function improvement, their effects are limited when mitochondria undergo irreversible alterations under septic challenge. Therefore, application of more effective approaches such as mitochondrial transplantation has been suggested. This review highlights the crucial role of mitochondrial damage in sepsis-related myocardial dysfunction, then provides an overview on mitochondrial-based therapies and current approaches to mitochondrial transplantation as a novel strategy, and proposes future directions for more researches in this field.

## 1. Introduction

Sepsis, as a major public health concern, is responsible for 50–60% of all deaths in intensive care units [1]. Sepsis causes acute lung damage, circulatory failure, and multiple-organ damage due to dysregulated host response to the infection [2, 3]. Intensive researches on sepsis have been of particular importance for decades. Given that most molecule-based therapies for sepsis have been failed in clinical setting, studies on pathological mechanisms involved in sepsis and finding better therapeutic targets are urgently needed [4, 5].

Myocardial dysfunction, often called septic cardiomyopathy, is a common manifestation in 40% of septic patients. The mortality in septic patients with myocardial dysfunction is up to 70% [6, 7]. Myocardial dysfunction in sepsis is mediated by several pathophysiological mechanisms that appear to be interrelated. Among these, mitochondrial dysfunction has attracted the attention of many researchers [8]. Several studies have reported that sepsis-related cardiac dysfunction is ameliorated by restoring mitochondrial function; however, impaired mitochondrial function worsens this condition [9]. It has been shown that overproduction of mitochondrial reactive oxygen species (ROS) and nitric oxide (NO), increased mitochondrial uncoupling, and the opening of mitochondrial permeability transition pore (mPTP) are involved in mitochondrial dysfunction in septic heart [10]. The results of preclinical and clinical researches have shown that there is a clear association between the degree of mitochondrial dysfunction and mortality in sepsis [11]. Investigating the important role of mitochondria in the pathophysiology of sepsis-related organ dysfunction can improve the diagnosis and monitoring of septic patients and provide more effective treatment approaches to improve patients' survival [12]. Since sepsis-related cardiac dysfunction and poor outcome of septic patients are associated with persistent mitochondrial dysfunction, it seems that removal of damaged mitochondria and production of healthy mitochondria can improve heart function in sepsis [9]. Mitochondrial-based therapies are potential approaches in sepsis treatment. Several experimental studies have examined the positive impacts of strategies targeting mitochondrial dysfunction in sepsis-related myocardial dysfunction and provided a potent basis for clinical studies [13, 14]. Although various therapeutic strategies have been used for improving mitochondrial function, their effects are limited when mitochondria undergo irreversible damage under septic challenge [15]. Therefore, application of other approaches such as mitochondrial transplantation has been suggested.

Mitochondrial transplantation is a therapeutic approach to repair or replenish damaged mitochondria via injecting healthy autologous harvested mitochondria from non-ischemic skeletal muscle into the injured organ. The physiological properties of healthy mitochondria provide the opportunity to replace damaged mitochondria and may protect the cells against further damage [16]. Transplanted mitochondria lead to the restoration of mitochondrial viability and function as well as improvement of postischemic cardiac function through mechanisms such as DNA repair, high-energy synthesis, and proteomic and transcriptomic alterations [17]. Based on the fact that elimination of dysfunctional mitochondria and production of healthy mitochondria improve the recovery of organ function in sepsis [9], application of mitochondrial transplantation can be considered as a promising treatment opportunity to improve sepsis-related organ dysfunction. Given that few studies have evaluated the effects of mitochondrial transplantation in sepsis-related organ dysfunction [18–20], it is important to perform extensive studies in this field.

This review outlines some key concepts regarding sepsis-related cardiac dysfunction and its associated mechanisms, and highlights the role of mitochondrial dysfunction in this context. This review also provides an overview on mitochondrial-based therapies in sepsis-related myocardial dysfunction and presents current approaches to mitochondrial transplantation as a promising strategy, and then proposes future directions for more researches in this field.

## 2. Sepsis-Related Myocardial Dysfunction

The mechanistic basis of cardiac dysfunction in sepsis is still under discussion. Early theories of sepsis-related heart dysfunction were based on the “global myocardial ischemia” hypothesis. But this hypothesis has been dismissed by laboratory and clinical studies showing a decrease in cardiac oxygen utilization and high coronary flow in sepsis [21]. Also, it seems that sepsis-induced cardiac dysfunction is an acute and reversible response similar to a self-protection response in which functional alterations rather than structural abnormalities are involved in its pathophysiology [1]. One theory suggests that sepsis-induced cardiac depression may have a cardioprotective impact on the heart similar to the “cardiac hibernation,” a phenomenon in the heart following ischemia that leads to the adaptive downregulation of cardiac function and oxidative metabolism, in which a protective adaptation is exhibited through attenuation of cellular energy expenditure in the myocardium. In other words, although cardiac suppression is occasionally lethal under septic condition, moderate sepsis causes myocardial hibernation [22, 23]. Many of the changes in cardiac mitochondria during sepsis appear to be a protective response rather than a purely pathological process. In this metabolic suppression, several mediators are involved such as inflammatory mediators, hormones, and gaseous signaling molecules (hydrogen sulfide, CO, and NO). These endogenous gases are involved in activating numerous transcription factors that participate in the regulation of mitochondrial genes expression such as hypoxia-inducible factor 1 (HIF-1) or nuclear factor erythroid 2-related factor 2 (Nrf2), as well as inhibiting complex IV, one of the oxidative phosphorylation (OXPHOS) complexes in the inner mitochondrial membrane [24]. In general, diminished mitochondrial function may be an adaptive mechanism in response to various degrees of tissue hypoxia and hypoperfusion, which is known as “mitochondrial hibernation.” Mitochondrial hibernation is associated with cellular downregulation of nonessential functions and reduced global rate of oxygen and adenosine triphosphate (ATP) consumption [25].

Based on the echocardiographic studies, defects in left ventricular systolic and diastolic functions have been observed in patients with sepsis. According to the experimental and clinical studies, reduced contractility and defective cardiac compliance under septic challenge are two important factors that lead to the cardiac dysfunction. Although there are structural and functional differences between right and left ventricles, similar functional changes have been detected in right ventricle, indicating that sepsis-associated right ventricular dysfunction closely parallels left ventricular dysfunction. But the relative role of the right ventricle in sepsis-induced cardiomyopathy is still unclear [26]. It has been shown that cardiomyocyte damage and increased troponins levels occur in sepsis [27, 28]. Circulating cardiac depressant factors such as tumor necrosis factor *α* (TNF-*α*), interleukin (IL)-6, IL-1*β*, NO, bacterial RNA and DNA, and lysozyme C have also been described in sepsis [28]. Two potential mechanisms involved in cardiac depression at the cellular level include overproduction of NO and derangements in calcium physiology [28]. Also, important changes in cardiovascular dysfunction under septic challenge include decreased ejection fraction and increased end-diastolic volume index. The association between left ventricular dysfunction and increased cardiotroponin (cTn) I and T in sepsis appears to be close and correlates with hemodynamic changes. Increased cTn levels are also associated with increased severity and poor short-term prognosis of sepsis [29]. It has been shown that intrinsic and extrinsic mechanisms are involved in hemodynamic collapse. The intrinsic mechanisms include proinflammatory mediators released by host immune cells, and extrinsic mechanisms include viruses, fungi, endotoxins, and other toxins. Both mechanisms induce a cascade of cellular events and release anti-inflammatory and proinflammatory mediators [30, 31]. Because the heart is a vital organ, compensatory mechanisms following sepsis preserve cardiac performance and thus mask the functional characteristics of cellular injury. Accordingly, cardiac dysfunction in septic challenge probably and locally occurs at the molecular level, and it is necessary to know the molecular and cellular mechanisms involved in sepsis-related cardiac dysfunction [32].

The mechanisms of sepsis-related heart dysfunction appear to be multifactorial. Our knowledge about the role of multiple mechanisms such as circulating cardiac suppressant factors, calcium, NO, apoptosis, and mitochondrial dysfunction in the development of sepsis-associated myocardial dysfunction has been improved in recent years. Since sepsis affects mitochondrial function, the intensity of mitochondrial dysfunction is associated with sepsis outcomes. Experimental and clinical studies have shown that ultrastructural abnormalities in cardiac mitochondria occur during sepsis [33]. Also, increased production of NO and superoxide levels by cardiac mitochondria during sepsis inhibits OXPHOS, decreases ATP generation, and leads to “cytopathic hypoxia,” an impairment in cellular oxygen utilization and ATP production, which is a key step in the development of sepsis-induced organ failure [34].

## 3. Crucial Role of Mitochondrial Dysfunction in Sepsis

Given that the heart requires continuous ATP delivery in order to preserve contractile function, mitochondrial dysfunction has detrimental effects on the heart. Mitochondrial dysfunction and energy depletion appear to be associated with myocardial dysfunction in sepsis [10]. Reports have shown that cytopathic hypoxia plays a more fundamental role in developing sepsis-related organ dysfunction than inadequate oxygen delivery. Given that mitochondria are the primary consumers of cellular oxygen and comprise 30% of myocardial volume, it seems that mitochondrial dysfunction plays a central role in cytopathic hypoxia [35], and mitochondria can be considered as an optimal therapeutic target in sepsis. Experimental studies have shown that sepsis leads to impaired mitochondrial structure and function, and overproduction of mitochondria-derived danger-associated molecular patterns (DAMPs) such as ROS, fragmented mitochondrial DNA (mtDNA), cardiolipin, N-formyl peptides, mitochondrial transcription factor A (TFAM), ATP, and cytochrome c, which worsen myocardial inflammation and sequential cardiomyopathy [36]. Numerous studies have examined the contribution of mitochondrial dysfunction in sepsis and have achieved conflicting results. Experimental studies evaluating the activity of respiratory enzyme within a few hours after sepsis induction demonstrated that no changes occur in the activity, some have reported an elevation, and others have shown depression in the activity [3, 14, 37]. Studies in models with sufficient duration and intensity of the sepsis often reported depression in the activity, indicating that a duration of at least 6 hours is often required for detecting impaired activity [38, 39]. A better and more accurate understanding of the mechanisms of mitochondrial dysfunction in sepsis can help to achieve new therapeutic targets to reduce organ dysfunction and mortality [40].

The exact cause of mitochondrial dysfunction in sepsis is not yet fully understood, but studies have shown that carbon monoxide, NO, and reactive oxygen/nitrogen species can directly disrupt a number of components of mitochondrial electron transport chain (ETC) complexes and mitochondrial respiration. Sepsis in lower metabolic rates is associated with reduced mtDNA and expression of major components of ETC complexes [24, 41]. Also, decreased expression of pyruvate dehydrogenase occurs in sepsis [42]. Mitochondrial dysfunction in sepsis induces failure in cellular energetic, which is a main reason for poor outcomes in patients [30]. Moderate activation of mitochondrial pathways in sepsis appears to be protective but, when left unchecked without recovery, contributes to the transition from adaptive to maladaptive organ dysfunction, indicating that therapeutic strategies should avoid abrogation of the protective influences [24]. There are several possible mechanisms involved in mitochondrial dysfunction in sepsis, which are described in the following section.

### 3.1. Mitochondrial Structural Damage

Normal mitochondrial function depends on mitochondrial complex ultrastructure. The cristae are home to important mitochondrial proteins, and their shape determines the efficiency of OXPHOS reactions, the surface area of the inner membrane, and the diffusion of solutes [43]. There is an association between the structure and function of mitochondria in healthy and diseased conditions. Mitochondrial morphological abnormalities such as cleared or condensed matrix, swelling, myelin figures, cristae abnormalities, internal vesicles, and mitochondrial membranes disruption have been proven in experimental studies of sepsis [10]. The main reason for mitochondrial swelling is damage to the inner mitochondrial membrane due to oxidative stress and calcium overload in the mitochondrial matrix, which enhances the permeability of the membrane pore and thus changes the osmotic pressure between the inner and outer membranes. Under mitophagy process, dysfunctional mitochondria are selectively targeted by autophagosomes and delivered to lysosomes for recycling or clearance [24, 41]. For sepsis recovery, mitochondrial homeostasis via balancing biogenesis and mitophagy is essential [44]. The important steps in restoring energy production and metabolism under sepsis recovery are activating biogenesis and mitophagy (for reservation of mitochondrial dynamic homeostasis) and increasing mitophagosomes and mitochondrial mass with various shapes [45].

### 3.2. Mitochondrial Oxidative Damage and Defective ETC and OXPHOS

In OXPHOS process, which occurs in the inner mitochondrial membrane, ATP production is mediated by the flow of electrons through the respiratory chain complexes. Electrons from FADH_2_ and NADH-H^+^ generated in the tricarboxylic acid (TCA) cycle are transferred from mitochondrial complexes, reoxidize carriers, and produce ATP [46]. ETC in the inner mitochondrial membrane consists of OXPHOS I–V complexes. Electrons are transferred from complex I to IV and produce ATP in complex V (ATP synthase) [30]. A key phenomenon in sepsis-related organ failure is OXPHOS abnormalities. These abnormalities are characterized with reduced oxygen consumption, ATP cellular content and synthesis, and mitochondrial membrane potential, decreased state 3 respiration (this state indicates the highest capacity of the respiratory chain itself in which saturating concentrations of adenosine diphosphate (ADP) and substrates are provided) and respiratory control ratio (this ratio indicates the coupling of phosphorylation to oxidation), reduced expression and/or activity of respiratory complexes, antioxidant capacity, and mtDNA content, increased state 4 respiration (an ADP-limited resting state in which ADP is depleted via its phosphorylation to ATP) or uncoupling, morphological abnormalities, and compromised biogenesis [24]. Mitochondrial function in sepsis is altered mainly due to ETC dysfunction. In sepsis, inflammatory mediators and reactive oxygen/nitrogen species directly cause defects in several components of the mitochondrial ETC complexes and respiration. Suppression of ETC complex activity occurs due to ROS accumulation in the mitochondrial matrix. ETC dysfunction leads to decreased ATP generation and overproduction of ROS as the results of defective OXPHOS process. ROS accumulation in mitochondria may impair ETC function and membrane permeability and induce calcium reflux and cytochrome c releasing. On the other hand, ROS releasing into the cytoplasm or extracellular space can lead to severe organ damage. In addition to OXPHOS in mitochondria, changes occur in intracellular nutrient metabolism progress such as glycolysis, fatty acid oxidation, and glutaminolysis [41].

ROS are a major source of mitochondrial damage and are considered as by-products of energy generation. In pathological conditions, accumulation of ROS occurs secondary to an imbalance between ROS scavenging and production and causes functional defects in DNA, lipids, and proteins through structural modifications [47]. Since mitochondria are considered as major producers of ROS, ETC is prone to the harmful effects of ROS, so increased production of mitochondrial ROS in sepsis leads to a vicious cycle to increase mitochondrial injury. The contribution of NO in this phenomenon is under discussion, as several studies have reported that NO is a mitochondrial toxin, while other studies have reported that NO has a protective role, suggesting that laboratory conditions, cell type, and NO source and concentration should be considered [48]. Mitochondrial function during sepsis is affected by NO. In sepsis, NO production is increased mainly by inducible nitric oxide synthase (iNOS) upregulation. This elevation in NO level is coupled with increased production of superoxide by the mitochondria, which favors the formation of peroxynitrite ions that are involved in modulating complex I in ETC, leading to the decreased cellular energy, respiratory inhibition, and a lack of cellular function in the heart and skeletal muscle [49]. Proximal inhibition of ETC appears to be protective and leads to a reduction in mitochondrial ROS formation via diversion of electrons into the Q-cycle by complex II [49, 50]. Under septic condition, enhanced expression of iNOS in vascular endothelial and smooth muscle cells, subsequent releasing of large amounts of NO, and a reduction in vascular tone occur. NO can bind to enzymes and lead to cellular dysfunction as well as inhibition of mitochondrial function directly or through interacting with free radicals. The combination of ROS and NO may lead to the formation of highly reactive peroxynitrite, enzymatic dysfunction, cell membrane injury, and mitochondrial dysfunction [3, 51]. Overproduction of ROS, NO, and other inflammatory factors can directly inhibit mitochondrial respiration. Competition of NO with oxygen for binding to cytochrome oxidase (complex IV) decreases enzyme activity, blocks ETC, and causes overproduction of superoxide. The reaction between superoxide and NO produces peroxynitrite and other nitrogen species that can modify the structure and performance of several mitochondrial proteins, especially complex I [52]. In sepsis, elevated superoxide dismutase (SOD)/catalase (CAT) ratio and the accumulation of H_2_O_2_ in the cell, decreased glutathione levels, and enhanced protein carbonyl groups and malondialdehyde levels occur. Alteration in the ratio of SOD/CAT such as SOD overexpression or CAT inhibition increases oxidative stress and morbidity under septic challenge [30].

In several experimental studies of sepsis, a decrease in mitochondrial state 3 respiration which indicates ETC inhibition, and in other studies, an increase in state 4 respiration which indicates abnormal permeability of inner mitochondrial membrane have been reported [50]. In addition, proinflammatory mediators can directly disrupt the function of respiratory chain complexes, leading to structural injury to mitochondrial DNA, proteins, and lipids, and defects in ATP production. Also, the activity of xanthine oxidase in plasma and the levels of NO, nitrite, and nitrate are increased. The antioxidative capacity in serum is decreased, while the levels of lipid peroxides, nitrotyrosine, and thiobarbituric acid reactive substances are elevated. Thus, sepsis is associated with enhanced oxidative stress [12].

### 3.3. Mitochondrial Calcium Homeostasis

The abnormalities in the intracellular calcium homeostasis have been studied in septic heart. A decrease in cytosolic calcium transients in most sepsis models is associated with increased diastolic cytoplasmic calcium and decreased content of sarcoplasmic reticulum (SR) calcium. Dysfunctional SR calcium transporters, especially leaky ryanodine receptors and the sarco/endoplasmic reticulum calcium-ATPase (SERCA), produce an enhanced release and a reduced reuptake of calcium, respectively. Despite significant studies suggesting the contribution of intracellular calcium imbalance in the pathogenesis of sepsis-induced heart dysfunction, few studies have specifically investigated the contribution of mitochondrial calcium [53, 54].

### 3.4. Mitochondrial Dynamics

**Figure 1:**
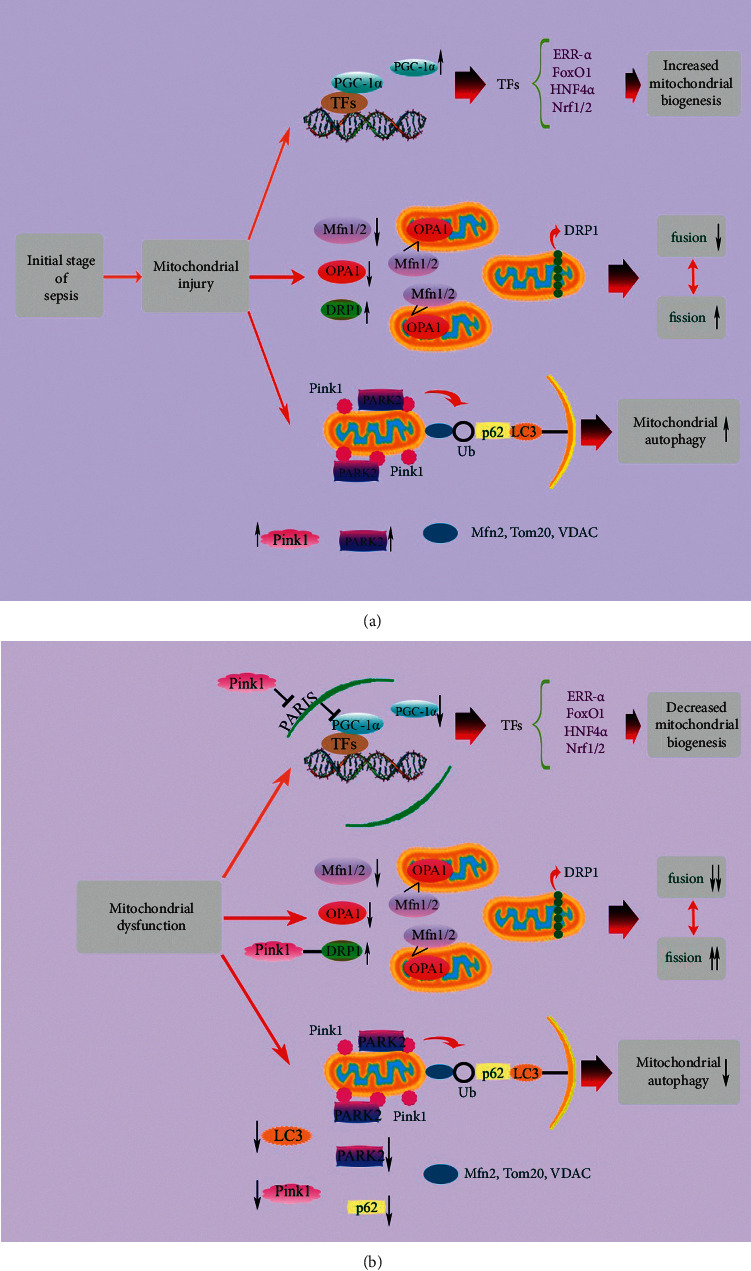
Mitochondrial quality control mechanisms under septic challenge. The upregulation of PGC-1*α* and transcription factors in the initial stage of sepsis promotes mitochondrial biogenesis. Decreased Mfn1/2 and OPA1 and increased DRP1 expression levels shift the balance of mitochondrial fusion and fission toward mitochondrial fission. Pink1 accumulation on mitochondria causes PARK2 translocation from cytoplasm into mitochondrial outer membrane and activation of mitophagy (a). With sepsis development, damaged mitochondria cannot be recovered and turn into mitochondrial dysfunction. The downregulation of PGC-1*α* and transcription factors prevents mitochondrial biogenesis. Persistent decreased expression levels of Mfn1/2 and OPA1 and increased expression level of DRP1 lead to mitochondrial fragmentation. Besides, decreased expression levels of Pink1, PARK2, p62, and LC3 lead to mitophagy deficiency (b). DRP1: dynamin-related protein 1; ERR-*α*: estrogen-related receptor *α*; FoxO1: forkhead box class-O; HNF4*α*: hepatocyte nuclear factor 4*α*; LC3: microtubule-associated protein 1 light chain 3; Mfn1/2: mitofusin 1/2; Nrf1/2: nuclear factor erythroid 2-related factor 1/2; OPA1: optic atrophy 1; PGC-1*α*: peroxisome proliferator-activated receptor *γ* coactivator 1*α*; Pink1: PTEN-induced putative protein kinase 1; TFs: transcription factors; Tom20: translocase of outer membrane; Ub: ubiquitin chain; VDAC: voltage-dependent anion channel.

Mitochondria are highly dynamic organelles that frequently experience fusion and fission to modulate their size, number, and morphology. In sepsis, mitochondrial dysfunction leads to the activation of mitochondrial fission and inactivation of mitochondrial fusion, which are involved in dysfunctional mitochondrial fragmentation and organ failure (Figure 1). Mitochondrial fragmentation causes Bcl-2 associated x protein (Bax) activation, outer membrane permeabilization, cristae remodeling, enhanced ROS generation, and ATP depletion, leading to the cell death and organ failure. Mitochondrial fragmentation and the expression of dynamin-related protein 1 (DRP1) are increased in skeletal muscle under septic challenge [55]. A study showed that, following treatment with lipopolysaccharide (LPS), a decrease in mitofusin 2 (Mfn2) at 4–6 hours and an increase in mitochondrial fragmentation at 6 hours occur in the liver. In addition, following cecal ligation and puncture (CLP) surgery, a decrease in Mfn2 at 12–18 hours, and an increase in DRP1 at 4 hours occur in the liver [56]. Moreover, it has been reported that TNF-*α* induces mitochondrial DRP1 expression and increases mitochondrial fragmentation and phosphorylated DRP1 levels in H9C2 cardiomyocytes [57].

### 3.5. Mitochondrial Biogenesis

Mitochondrial biogenesis means mitochondrial growth and division that requires appropriate functioning of cellular processes such as replication of mtDNA, the processes of fusion-fission, coordinated synthesis and import of nuclear genome-encoded proteins, and synthesis of mitochondrial genome-encoded proteins. In the initial stage of sepsis, elimination of injured mitochondria is compensated by the production of new mitochondria through enhancing mitochondrial biogenesis, which improves survival and reverses organ damage; however, inhibition of mitochondrial biogenesis during sepsis development aggravates outcomes (Figure 1). The adaptive versus maladaptive impact of mitochondrial biogenesis activation in sepsis is still under discussion. Defective mitochondrial energetics may be involved in myocardial dysfunction, and mitochondrial biogenesis activation may display beneficial impacts in endotoxemia [10, 58]. Activation of mitochondrial biogenesis in sepsis-induced inflammation may adjust mitochondrial mass, distribution, and functionality in order to restore the population of functional mitochondria in the heart. Maintaining the population of functional mitochondria via balancing mitochondrial biogenesis and turnover through mitophagy is required for the survival and normal function of the cell [59].

A variety of cellular stressors such as oxidative stress, increased NO, and elevated adenosine monophosphate (AMP)/ATP ratio are involved in triggering mitochondrial biogenesis, leading to the activation of peroxisome proliferator-activated receptor *γ* (PPAR*γ*) coactivator (PGC) family, especially PGC-1*α* and PGC-1*β* [60]. Mitochondrial biogenesis is regulated by several molecular signals such as TFAM, Nrf1/2, and PGC-1*α* in response to energy demand, and is involved in increasing mitochondrial mass and the recovery of mitochondrial network [61, 62]. PGC-1*α* plays a role in coactivating and/or increasing the expression of transcription factors such as Nrf1/2, estrogen-related receptor *α* (ERR)*α*/*γ*, and TFAM, that are involved in mediating the transcription of nuclear and mitochondrially encoded OXPHOS subunits, OXPHOS assembly factors, nuclear proteins essential for transcription and replication of mtDNA, and mitochondrial protein import components [60, 63]. In addition, PGC-1*α*/*β* is involved in stimulating the expression of genes encoding proteins and enzymes of fatty acid oxidation in the heart [64]. In the initial stage of sepsis, increased inflammatory cytokines (TNF-*α*, IL-6, and IL-1*β*) lead to an inflammatory cytokine storm, which is a main cause of death in this stage [9]. Mechanisms linking the activation of PGC-1*α* to the downregulation of inflammation genes may be associated with recovery of mitochondrial function and reduced ROS production by PGC-1*α* activation [65]. Studies have shown that therapeutic strategies targeting mitochondrial biogenesis may reverse sepsis-induced organ failure [9].

### 3.6. Mitochondrial Autophagy

**Figure 2:**
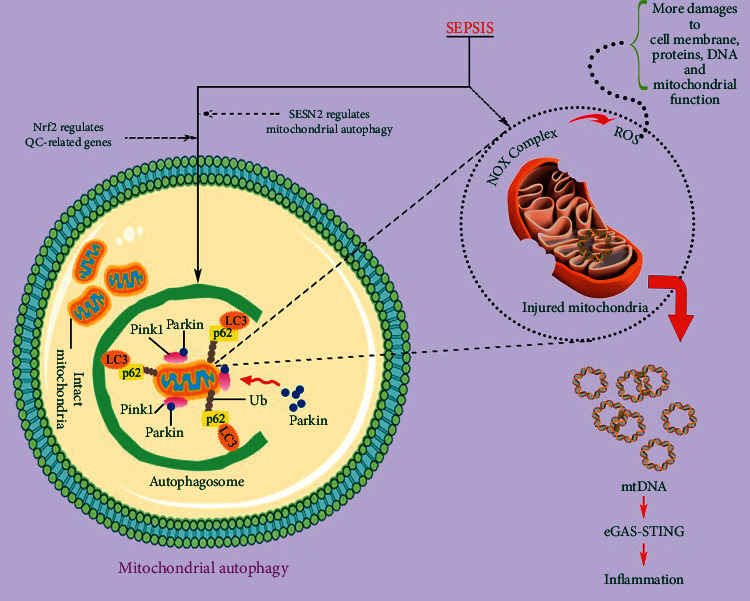
Oxidative stress and mitophagy in damaged mitochondria. The number of damaged mitochondria increases in the initial stage of sepsis. Damaged mitochondria lead to mitochondrial dysfunction via ROS signaling pathways and mtDNA. Autophagy removes damaged mitochondria. Cytokines such as SESN2 and Nrf2 regulate autophagy process. cGAS: cyclic GMP-AMP synthase; LC3: microtubule-associated protein 1 light chain 3; mtDNA: mitochondrial DNA; NOX: NADPH oxidase; Nrf2: nuclear factor erythroid 2-related factor 2; Pink1: PTEN-induced putative protein kinase 1; QC: quality control; ROS: reactive oxygen species; SESN2: sestrin 2; STING: interferon gene stimulator; Ub: ubiquitin chain.

Autophagy is involved in selecting damaged mitochondria for elimination via autophagy-related genes- (ATG-) dependent or independent pathways, leading to the renewal of damaged organelles and proteins through lysosomal-dependent degradation pathways to counteract oxidative stress [66]. Autophagy plays a central role in the pathology of organ dysfunction caused by sepsis. Autophagy is altered dynamically during the progression of sepsis. In the later stage of sepsis, inadequate and maladaptive autophagy occurs, which is associated with upregulation of mammalian target of rapamycin (mTOR) signaling. This ineffectiveness elimination of toxic substances and damaged organelles leads to the accumulation of DAMPs [67]. Oxidative injury may lead to apoptosis and worsen sepsis-related myocardial injury. Induction of autophagy and replacing or repairing damaged mitochondria are essential in order to maintain basal function of the cell, prevent oxidative injury, and improve sepsis-induced heart damage [67]. Elimination of damaged mitochondria is done through mitophagy, which is considered as a part of the mitochondrial quality control system to cope with oxidative stress and remove damaged mitochondria in sepsis development (Figure 2). Recovery of septic organ function depends on the elimination of damaged mitochondria and the production of functional mitochondria that appear to be mediated by stimulating mitochondrial biogenesis [67].

There are several pathways for identification and elimination of dysfunctional mitochondria. Pink1/Parkin pathway (PARK2) which is regulated by PTEN-induced putative kinase 1 (Pink1) and E3 ubiquitin ligase Parkin targets mitochondria with lost membrane potential and delivers them to the autophagosomes for degradation. In other words, this pathway is involved in ubiquitinylation of mitochondrial specific substrates that can be recognized by p62, which binds to microtubule-associated protein 1 light chain 3 (LC3) [68]. In sepsis, mitochondria are susceptible to injury, and the reduction of inner membrane potential is observed. Consequently, Pink1 accumulation in the outer membrane of the mitochondria and Parkin recruitment onto the mitochondria and its activation by Pink1 occur. Activated Parkin then contributes to the building of ubiquitin chains on injured mitochondria to tag them for degradation. The ubiquitin chain binds to the autophagic protein receptor, which binds to LC3 on the autophagosome for mitophagy induction [67, 69]. SQSTM1/p62 is involved in the identification of ubiquitinated mitochondrial proteins and their assembly into autophagosomes. Isolation of damaged mitochondria by autophagosomes and their degradation through fusion with lysosomes have been detected in the recovery of organ function under sepsis [70, 71]. The ubiquitin-independent pathway promotes direct binding of autophagy-related protein 8 (ATG8) family proteins to autophagy receptors including Nip3-like protein X (NIX) [72, 73].

Beclin-1 is involved in the promotion of mitochondrial biogenesis through Pink1/Parkin and AMP-activated protein kinase (AMPK)/unc-51 like autophagy activating kinase 1 (ULK1) pathways and selectively facilitates Pink1-Parkin mitophagy to eliminate damaged mitochondria [67]. Beclin-1 stimulates mitophagy in response to LPS, which is accompanied with selective increase in the recruitment of Pink1 and Parkin to the mitochondria and strong suppression of the receptor proteins Bcl-2/adenovirus E1B-19 KD interacting protein 3-like (BNIP3L) and BNIP3. This means that Beclin-1 has protective impact on mitochondria by selectively activating a particular mitophagy pathway rather than inducing all types of mitophagy [74]. In general, increased Beclin-1 signal in sepsis maintains the mass of functional mitochondria via selective facilitation of Pink1-Parkin mitophagy to eliminate dysfunctional mitochondria and promote mitochondrial biogenesis through Pink1/Parkin and AMPK/ULK1 pathways. But when Beclin-1 signal is not sufficiently available under septic condition, stronger mitochondrial BNIP3 signal and low activities of ULK1 and AMPK together with inflammatory responses worsen mitochondrial damage [36].

Effective removal of unwanted mitochondrial molecules and damaged mitochondria is essential to preserve healthy myocardium. Mitophagy may remove damaged mitochondria before they cause cell injury or death but may also act as a survival pathway through suppression of apoptosis, or as a back-up mechanism when apoptotic process is defective [10]. Under physiological conditions or mild stress, autophagy is adaptive and provides cellular quality control for promoting survival. But under chronic or severe stress conditions, insufficient or severe autophagy leads to the accumulation of toxic substances or excessive self-degradation, respectively, both of which are maladaptive and lead to the cell death [75, 76]. Increased autophagy in several organs such as the heart has been identified in experimental models of sepsis, but the mechanisms of its occurrence are still unclear [77]. It is not clear whether autophagy in sepsis is a process to eliminate damaged mitochondria or is part of a cellular program that leads to apoptosis. Further researches are required to explain the role of mitophagy in myocardial dysfunction under septic challenge and to determine how mitophagy may directly regulate mitochondrial biogenesis [36]. Both adaptive and maladaptive properties of autophagy in the heart under septic condition have been observed in previous studies. Several studies have shown that pharmacological stimulation of autophagy protects the heart, so autophagy is an adaptive response. In contrast, decreased autophagy activity using its inhibitors or antioxidants has been shown to improve cardiac contractility in the LPS-induced endotoxemia, indicating maladaptive autophagy [78]. The discrepancy of these results is due to the differences in laboratory setting such as sepsis severity, drug specificity, and timing of delivery. In details, with small doses of LPS and mild sepsis, autophagy increases in proportion to the insult magnitude. But in severe sepsis, a decrease in capability occurs proportionally to the severity of the insult. It seems that, in mild sepsis or the beginning of the sepsis, autophagy is adaptive and is involved in maintaining normal function of the organs and promoting survival. But in severe sepsis, autophagic response is insufficient and leads to maladaptive outcome [36].

### 3.7. Mitochondrial Permeability Transition Pore (mPTP)

Under septic challenge, mitochondrial calcium overload can be a key mechanism of mitochondrial dysfunction in the heart [79]. Low survival, cardiac dysfunction, increased state 4 and decreased state 3 respiration, and enhanced opening of mPTP have also been observed during sepsis. The primary trigger for mPTP opening is calcium overload, but pore sensitivity depends on oxidative stress, depletion of adenine nucleotide, increased inorganic phosphate, and mitochondrial depolarization [80]. The consequences of mPTP opening includereduction of state 3 respiration, loss of mitochondrial membrane potential, rupture of the mitochondrial outer membrane, mitochondrial swelling, and activation of proapoptotic pathways and necrotic cell death. Inhibition of mPTP by treatment with cyclosporine A causes improved cardiac performance and survival and decreased mitochondrial dysfunction [81].

### 3.8. Mitochondrial Apoptosis

**Figure 3:**
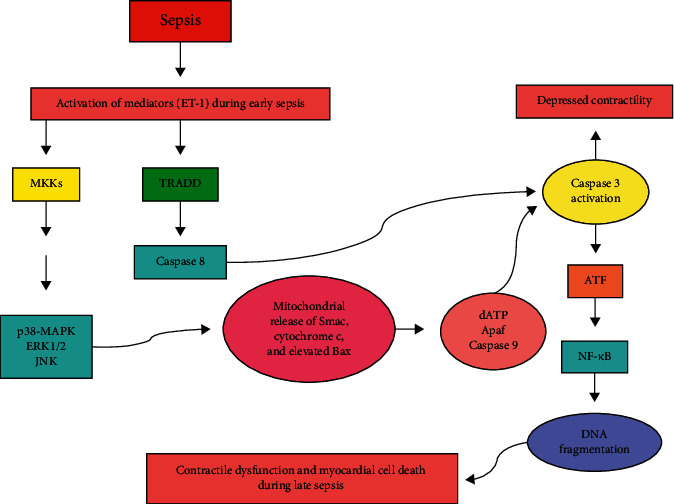
Cellular events involved in the induction of apoptosis through activation of extrinsic and intrinsic pathways in sepsis. See the text for detailed information. Apaf: apoptosis protease activating factor-1; ATF: activating transcription factor 1 and/or 2; Bax: Bcl-2 associated x protein; ERK1/2: extracellular signal-regulated kinase 1/2; ET-1: endothelin-1; JNK: c-Jun N-terminal kinase; NF-*κ*B: nuclear factor *κ*B; p38-MAPK: p38-mitogen-activated protein kinase; Smac: second mitochondria-derived activator of caspases; TRADD: TNF receptor-associated death domain.

Apoptosis occurs through extrinsic and intrinsic signaling pathways. Both pathways appear to be activated and regulated by mitochondrial events in sepsis (Figure 3). In sepsis, activation of extrinsic apoptotic pathway occurs through the TNF receptor-associated death domain (TRADD) receptors. Activation of death receptor causes caspase-8 activation, which then leads to the cleavage and activation of other caspases to provoke cell death. Activation of intrinsic pathway as a result of chemicals and growth factor deprivation leads to the perturbation of mitochondria, proteins releasing such as second mitochondria-derived activator of caspases (Smac) and cytochrome c, and enhanced expression of proapoptotic Bax proteins. Activation of both pathways of apoptosis leads to the higher levels of activated caspase-3. There is a cross-talk between extrinsic and intrinsic pathways by proapoptotic B-cell lymphoma 2 (Bcl-2) proteins, especially BH3 interacting-domain death agonist (Bid) [32, 50].

Following 3 and 7 days of sepsis, an increase in the number of apoptotic cells has been detected, which is associated with increased expressions of Bax, TRADD, and active caspase-3 in the heart [82]. Also in septic heart, mitochondrial dysfunction occurs that is associated with increased active caspase-3 and myocardial dysfunction. Thus, the intrinsic pathway of apoptosis can play an important role in promoting sepsis-induced heart dysfunction [83]. Inappropriate regulation of apoptosis in immune cell may also be involved in immune dysfunction and organ failure in sepsis [84]. The impact of sepsis on apoptosis rate is cell and tissue-specific, and minimal cell death has been explored in vital organs, suggesting that cell death primarily cannot describe why organs fail in sepsis. However, mitochondrial apoptotic pathways are needed to remove damaged mitochondria and have been linked to sepsis-related organ failure [50].

## 4. Mitochondrial-Based Therapeutic Opportunities in Sepsis-Related Myocardial Dysfunction

One of the interesting approaches in sepsis treatment is mitochondrial-based therapies. Many experimental studies have targeted mitochondrial dysfunction in septic heart and provided valuable results in this regard, which are described in the following section. By addressing these interesting studies, we aimed to elucidate the way for developing more effective therapeutic strategies to improve outcomes in septic patients in the future.

### 4.1. Targeting Mitochondrial Matrix and Respiratory Chain

#### 4.1.1. MitoQ

Mitochondrial antioxidant system is overwhelmed under septic challenge. Antioxidants targeted into the mitochondria have shown more protective effects than nontargeted antioxidants. Conjugation of an antioxidant into a lipophilic cation triphenylphosphonium (TPP) that concentrates in the matrix has been examined as a technique to achieve this approach [85]. MitoQ, which includes ubiquinol antioxidant (or coenzyme Q10) conjugated to TPP, is a potent scavenger of free radicals with anti-inflammatory and antioxidative properties. MitoQ has been studied more than other antioxidants and had beneficial effects in sepsis models. A study showed that endotoxin-induced sepsis impaired mitochondrial function, increased the levels of oxidative stress biomarkers, decreased cardiac pressure-generating capacity, and activated intrinsic pathway of apoptosis in rat and mice hearts. Administration of MitoQ prevented cardiac mitochondrial damage, caspase-3 and caspase-9 activation, and endotoxin-induced reduction in cardiac pressure-generating capacity, and also ameliorated increased level of protein carbonyl, an index of tissue free radical generation [86]. Another study in a lipopolysaccharide-peptidoglycan G (LPS/PepG) model of sepsis with organ dysfunction in rats showed that treatment with MitoQ reduced organs dysfunction (as evidenced by reduced levels of biochemical markers of acute renal and liver damages) and augmented mitochondrial membrane potential. In addition, *in vitro* study of sepsis in endothelial cells showed that administration of MitoQ reduced oxidative stress and protected mitochondria from injury by reducing ROS production and maintaining mitochondrial membrane potential [87].

#### 4.1.2. Mitochondria-Targeted Vitamin E (Mito-Vit-E)

In a pneumonia-related sepsis model in Sprague–Dawley rats, administration of single dose of Mito-Vit-E improved mitochondrial antioxidant defense, protected mitochondria from oxidative injury, improved mitochondrial respiratory function, and prevented mitochondrial membrane injury in cardiomyocytes. Also, treatment with Mito-Vit-E enhanced total antioxidant capacity, reduced H_2_O_2_ production, and inhibited cytokine production and neutrophil infiltration. The anti-inflammatory effect of Mito-Vit-E was more efficient than conventional Vit-E at the same dose, which is distributed globally. These findings support a hypothesis that mitochondrial injury is associated with inflammatory response in the heart during sepsis, and mitochondrial antioxidants can be considered as a beneficial therapeutic target for the treatment of sepsis [88]. However, it has been reported that antioxidant conjugated to TPP could not be helpful in septic patients, because mitochondrial damage during sepsis decreases the concentration levels of such antioxidants in the mitochondria [89].

#### 4.1.3. Antioxidant Peptides

A class of cell-permeable small peptides were innovated by Szeto–Schiller and named SS peptides. These peptides selectively partition to the inner mitochondrial membrane and exert intrinsic protective impacts on the mitochondria. Selective targeting of these peptides into the mitochondria is due to their positive charge and the negative charge of cardiolipin in inner mitochondrial membrane. These peptides are very potent in decreasing mitochondrial ROS production, scavenging ROS, and preventing mitochondrial permeability transition, apoptosis, and necrosis induced by oxidative stress. Although there is a need for evaluating the beneficial effects of these peptides on sepsis-related cardiomyopathy, their positive effects have been recognized in oxidative stress models of ischemia/reperfusion (I/R) injury [90].

#### 4.1.4. Melatonin

Melatonin and its metabolites which accumulate in the mitochondria have potent antioxidative and anti-inflammatory activities in cell and mitochondria. Following CLP-induced sepsis in wild-type (iNOS^+/+^) mice, inhibition of respiratory chain complexes occurred, but there was no alteration in ETC activity during sepsis in iNOS-deficient (iNOS^−/-^) mice. This indicates that nitrosative/oxidative stress caused by induction of mitochondrial inducible nitric oxide synthase (i-mtNOS) is responsible for respiratory chain disturbance. Disturbed ETC reduces mitochondrial capacity in ATP synthesis and subsequently decreases cardiac contractility. But treatment with melatonin decreased iNOS expression, reduced i-mtNOS activity, increased ETC complexes activity, normalized lipid peroxidation level, and decreased free radicals. In brief, beside cytosolic iNOS, cardiac mitochondria have i-mtNOS which is induced during sepsis. Alongside sepsis-induced mitochondrial dysfunction, oxidative stress, and depletion of ATP synthesis, the level of i-mtNOS is also increased. These alternations can lead to myocardial dysfunction. However, treatment with melatonin in iNOS^+/+^ mice ameliorated mitochondrial dysfunction, normalized ATP production, and increased survival rate [91].

#### 4.1.5. Alpha-Lipoic Acid

In a sepsis model induced by CLP in rats, treatment with alpha-lipoic acid prevented oxidative stress, decreased NO generation, and improved mitochondrial function. The mechanism of alpha-lipoic acid activity is complicated, but it seems that modulation of glutathione and other thiols levels, production of antioxidants, and regulation of different signaling pathways are involved [92].

#### 4.1.6. Glutamine

The positive effects of glutamine in experimental sepsis have been proven. Under septic challenge, glutamine has anti-inflammatory properties and is able to improve lung function and increase survival [93]. A study revealed that the activity of cytochrome c oxidase (COX), a terminal oxidase in mitochondrial ETC, was decreased 24 hours after CLP. Intraperitoneal injection of glutamine restored the activity of COX and improved mitochondrial function, which were accompanied with significant increases in myocardial systolic and diastolic functions. Since ATP availability and TCA cycle mediators were not assessed in this study, it can be merely demonstrated that there is an association between improved myocardial performance following administration of glutamine and abrogation of cardiac COX inhibition, not cause. Additionally, it is possible that improved heart function following glutamine injection during sepsis may be independent of or unrelated to its influence on OXPHOS and COX activity, because COX activity was restored in the myocardium following glutamine injection during sepsis; however, cardiac performance did not reach the values in saline-injected controls. It seems that uncoupling effect of glutamine on mitochondria or extramitochondrial effects of glutamine may be involved in this scenario [94].

#### 4.1.7. Exogenous Cytochrome c

OXPHOS impairment is a cause of sepsis-related cardiac depression. It has been shown that intravenous injection of exogenous cytochrome c caused mitochondrial recovery, restored the levels of heme c, elevated the kinetic activity of COX, and improved cardiac function under septic condition [95].

#### 4.1.8. Caffeine

Caffeine (1,3,7-trimethylxanthine), a cyclic adenosine monophosphate- (cAMP-) phosphodiesterase inhibitor, increases the levels of intracellular cAMP and the activation of protein kinase A, and is capable to increase COX activity via enhancing cAMP levels [14]. The results of a study showed that CLP-induced sepsis in rats impaired COX activity in the myocardium; however, administration of caffeine following induction of sepsis restored COX activity, increased left ventricular pressure, and improved heart function. Taken together, caffeine led to OXPHOS stimulation under septic condition, which was associated with improved cardiac function and survival [96].

#### 4.1.9. Resveratrol

The severity of sepsis-related myocardial damage is associated with PGC-1*α* deficiency [97]. Smeding et al. demonstrated that CLP-induced sepsis in mice led to mitochondrial swelling, reduced translation, expression, and activity of PGC-1*α*, decreased mitochondrial integrity, and diminished cardiac contractility. However, treatment with resveratrol increased PGC-1*α* and improved myocardial contraction but had no effect on 48-hour mortality. Additionally, resveratrol enhanced the expression levels of genes involved in detoxification, oxidative stress management, and OXPHOS, preserved myocardial capacity in energy generation, prevented secondary damages, suppressed inflammation, and reversed sepsis-related cardiac remodeling [98]. Another study in an LPS-induced sepsis model in mice showed that administration of resveratrol significantly decreased proinflammatory cytokine production and increased Nrf2 activation in the myocardium, which were accompanied with diminished cardiac damage. Also, *in vitro* evaluation in cultured primary human cardiomyocytes showed that resveratrol caused activation of Nrf2, reduction of LPS-induced ROS production, and protection of cells against LPS-induced apoptosis. Nrf2 knockdown diminished the protective effects of resveratrol against LPS-induced cell death. The beneficial effects of resveratrol in reduction of LPS-induced myocardial toxicity could be associated with increased activity of Nrf2 antioxidant defense pathway [99].

#### 4.1.10. Magnesium Supplementation

Ahmed et al. showed that LPS injection in mice increased plasma levels of creatine kinase (CK)-MB, increased the levels of myocardial ADP, and reduced myocardial creatine phosphate, ATP, AMP, and ATP/ADP ratio. Moreover, LPS injection led to an increase in tissue thiobarbituric acid reactive substances and a decrease in reduced glutathione levels. Elevation of myocardial lactate : pyruvate ratio and attenuation of Na^+^, K^+^-ATPase activity were also observed in mice subjected to LPS injection. Also, ultrastructural evaluation showed severe damage to mitochondria including decreased matrix density, mitochondrial vacuolization and swelling, and disrupted integrity of cristae. Pretreatment with magnesium in both doses (20 or 40 mg/kg) led to the normalization of myocardial contents of ADP, AMP, and reduced glutathione. Pretreatment with magnesium in a higher dose (40 mg/kg) led to the normalization of plasma levels of CK-MB, the levels of creatine phosphate, and tissue thiobarbituric acid reactive substances, elevation of myocardial content of ATP, and restoration of normal Na^+^, K^+^-ATPase activity. Also, magnesium (20 mg/kg) normalized cardiac pyruvate content and decreased lactate accumulation and the ratio of lactate : pyruvate, but the higher dose exerted better effects. Mitochondrial ultrastructural evaluation showed that higher dose of magnesium caused better mitochondrial improvement. In general, it can be said that administration of magnesium could exert protective effects in LPS-induced cardiotoxicity [100].

### 4.2. Targeting Mitochondrial Membrane Stability

#### 4.2.1. Cyclosporine A

Cyclosporine A is an immunosuppressant drug that causes inhibition of mPTP via interacting with cyclophilin D. Larche et al. showed that CLP-induced sepsis led to impaired respiration efficacy of mitochondria and multiple organ dysfunction. This study revealed that under chronic sepsis, mPTP opening might be directly involved in multiple organ damage and mortality rate. Moreover, endotoxemia increased caspase-9- and caspase-3-like activities in the myocardium, which were associated with cardiac dysfunction. Treatment with cyclosporine analogs prevented sepsis-induced organ damage, decreased sepsis-related contractile dysfunction, and reduced mortality rate through inhibition of mPTP and caspase-3-like activity [81]. Another study indicated that mitochondrial damage during acute endotoxemia caused impairment in myocardial contractility, and calcineurin was involved in regulation of mitochondrial respiration, ROS generation, protein carbonylation, tissue nitration, and cardiac metabolism and contractile function. Nevertheless, the mechanism involved in the regulation of mitochondrial function by calcineurin during endotoxemia needs to be clarified. Pretreatment with cyclosporine A inhibited calcineurin-related signaling pathways, decreased NOS2 expression, and subsequently prevented NO-induced mitochondrial injury and cardiac dysfunction. But further researches are needed to determine whether calcineurin-mediated myocardial dysfunction and mitochondrial damage are adaptive or maladaptive under septic challenge [101].

### 4.3. Targeting Mitochondrial Biogenesis

#### 4.3.1. Exogenous Sodium Hydrosulfide (NaHS)

Liang et al. demonstrated that CLP-induced sepsis in mice led to mitochondrial swelling, reduced ATP levels, disrupted mitochondrial ridge, increased cTnI levels, and cardiac structural injury. Administration of exogenous NaHS, a hydrogen sulfide (H_2_S) donor, increased the levels of ATP and mRNA expression levels of TFAM, which were positively correlated with mRNA expression levels of PGC-1*α* and Nrf2. Altogether, administration of exogenous NaHS provided protective effect against myocardial mitochondrial damage in sepsis through upregulation of PGC-1*α* and Nrf2 expression levels and promotion of mitochondrial biogenesis by transcription of TFAM [102].

#### 4.3.2. Overexpression of Thioredoxin 1

In an experimental study, transgenic mice with thioredoxin 1 cardiac-specific overexpression were subjected to CLP surgery. Thioredoxin 1 overexpression increased survival, preserved myocardial contractile reserve, and attenuated myocardial damage, which were associated with preserved mitochondrial membrane potential and complex activities, increased PGC-1*α* gene expression, and extended antioxidant protection. Overall, thioredoxin 1 overexpression attenuated myocardial dysfunction and mitochondrial injury and activated mitochondrial turnover (biogenesis and mitophagy) under septic condition [103].

#### 4.3.3. Overexpression of Uncoupling Protein 2 (UCP2)

UCP2, a member of the uncoupling protein family, is localized on inner mitochondrial membrane. UCP2 ion carrier opening causes protons leakage through the mitochondrial intermembrane space into the mitochondrial matrix without ATP synthase. Overproduction of ROS activates UCP2 and regulates ROS generation via negative feedback. An *in vitro* study showed that LPS stimulation elevated the levels of lactate dehydrogenase (LDH), CK, TNF-*α*, and IL-6, decreased mitochondrial membrane potential, and increased mitochondrial flashes (mitoflashes) in cardiomyocytes. Mitoflashes are transient signal from transient bursts of ROS and alterations in pH that occur under certain physiological or pathological conditions. UCP2 overexpression in cardiomyocytes decreased the levels of LDH, CK, TNF-*α*, and IL-6, improved mitochondrial membrane potential, reduced the frequency of mitoflashes, and reversed LPS-induced cardiomyocytes damage. It can be said that overexpression of UCP2 is involved in protecting the heart against LPS-induced myocardial injury via regulation of mitoflash frequency [104]. Another study in LPS-induced cardiomyopathy showed that when UCP2 was upregulated, cytochrome c and caspase-3 were decreased, whereas Beclin-1 and LC3 were enhanced, indicating the position of UCP2 in crosstalk between apoptosis and autophagy. In addition, UCP2 blocking led to increased cytochrome c and caspase-3 and catastrophic damages in myocardial cells. Both *in vitro* and *in vivo* evaluations revealed that UCP2 had protective effects against LPS-induced cardiomyopathy via regulating the balance between apoptosis and autophagy in cardiomyocytes, and it may play a role in myocardial function homeostasis and cardiomyocytes activity [105]. Zheng et al. demonstrated that LPS/PepG-induced sepsis in H9C2 cells caused mitochondrial ultrastructural damage, mitochondrial membrane potential dissipation, excessive ROS production, mtDNA deletion, decreased ATP levels, increased UCP2 mRNA and protein expression, and elevated CK, LDH, TNF-*α*, and IL-6 levels. UCP2 silencing by small interfering RNA (siRNA) exacerbated damage in H9C2 cells and mitochondria, indicating that UCP2 may play a protective role in cardiomyocytes under septic challenge [106].

## 5. Mitochondrial Transplantation

### 5.1. Feasibility and Limitation

Although various strategies have been used for the improvement of mitochondrial function under septic challenge, their effects are limited due to sepsis-induced irreversible alterations in mitochondria. Therefore, application of mitochondrial transplantation is suggested. Autologous mitochondrial transplantation is a therapeutic strategy in which autologous intact mitochondria from non-ischemic skeletal muscle is transplanted into the damaged myocardium [107]. Previous reports have shown that transplanted mitochondria improve heart function following ischemia by recovering mitochondrial function through intracellular and extracellular mechanisms including transcriptomic and proteomic changes, high-energy synthesis, and DNA repair. The results of a study showed that mitochondrial transplantation in pediatric patients with myocardial I/R injury who required extracorporeal membrane oxygenation improved ventricular function in several days following transplantation. Also, mitochondrial transplantation had no adverse short-term complications including intramyocardial hematoma, scarring, and arrhythmia [17]. In a study on myocardial I/R model in rabbits, mitochondria were injected just before the reperfusion that is a critical period to salvage myocardium and restore heart function. Injection of respiration competent and viable mitochondria isolated from non-ischemic tissue into the ischemic region of heart tissue during early reperfusion decreased myocardial infarct size and the levels of CK-MB and cTnI, elevated postischemic functional recovery, and enhanced cellular viability in the ischemic region, which were associated with decreased caspase-3 activity and improved mitochondrial function [108]. In the clinically relevant swine model of myocardial I/R injury, Kaza et al. demonstrated that transplanted mitochondria remained in the myocardium for at least 4 weeks following injection and enhanced the viability of myocardial cell after I/R. The mechanisms involved in cardioprotective effects of mitochondrial transplantation include cell rescue, replacement of damaged mtDNA, energy synthesis, upregulation of mitochondrial protein pathways, production of precursor metabolites for energy and cellular respiration, and upregulation of cytokines involved in arteriogenesis, angiogenesis, immunomodulation, migration of progenitor cell, inhibition of apoptosis, and increased cell salvage. It should be noted that the source of mitochondria was not important in allowing for cardioprotection. Accordingly, total, interfibrillar, and subsarcolemmal mitochondria isolated from the liver, myocardium, and skeletal muscle exert similar cardioprotective effects [109]. Masuzawa et al. showed that transplantation of autologously derived mitochondria immediately prior to myocardial reperfusion in rabbits caused reduction of cTnI and CK-MB levels, normalization of myocardial contractile function, prevention of apoptosis in the ischemic region, reduction of inflammatory factors, elevation of oxygen consumption and the synthesis of high energy phosphate, and the induction of cytokine mediators and proteomic pathways involved in cardioprotection. Also, the transplanted mitochondria did not cause arrhythmia or auto-immunity [110].

Although mitochondrial transplantation approach has many advantages, this approach has some limitations. For example, various methods for mitochondrial delivery have been studied to improve the absorption and transplantation efficiency of mitochondria, but these methods have disadvantages and may interfere with the efficacy of transplantation. Thus, optimizing these methods according to the cell or tissue type and developing more efficient cell- or tissue-specific delivery techniques are required to avoid mitochondrial transplantation into unwanted tissues [111]. Moreover, due to the sensitivity of exogenous mitochondria and rapid reduction of their survivability and activity, mitochondrial isolation should be carried out at a low temperature within a short time. Besides, isolated mitochondria have to be used rapidly following isolation, because there are currently no appropriate techniques for long-term storage of isolated mitochondria, indicating that there is an immediate need for innovation of optimal protocols to isolate and store mitochondria that preserve the integrity of mitochondria for their application in clinical setting [15, 112]. Based on the above-mentioned limitations, it is crucial to resolve these challenges.

### 5.2. Mitochondrial Transplantation as a Promising Approach in Sepsis

Investigating the effects of mitochondrial transplantation in sepsis-related myocardial dysfunction is indeed a notable direction to head towards. But there are challenges in this way. If all challenges are elucidated and resolved effectively, the hypothesis of “mitochondrial transplantation as a treatment for sepsis-related myocardial dysfunction” will merit clinical examinations. In this regard, by reviewing some experimental evidences about the impact of mitochondrial transplantation on sepsis, we tried to make insights in this scenario and encourage researchers to exploit mitochondrial transplantation as an intriguing therapeutic strategy in sepsis-related organ dysfunction.

Hwang et al. [18] showed that injection of isolated mitochondria following cecal slurry-induced sepsis in rats caused survival improvement, bacterial clearance, alleviation of mitochondrial dysfunction and apoptosis, and reduction of both hyperinflammation and immune paralysis in the spleens. These positive effects of mitochondrial transplantation were also proven in the *in vitro* model of sepsis. Mitochondrial transplantation exerted immunosuppressive influence in the hyperinflammatory stage of sepsis and also showed an immune-enhancing influence in the late immune paralysis stage of sepsis. In addition, mitochondrial transplantation showed an antiapoptotic effect possibly via modulation of intrinsic pathway of apoptosis. In this study, mitochondrial transplantation decreased fission, which might decrease apoptosis. However, lower levels of Bcl-2 antagonist/killer 1 (Bak) and a little decrease in Bcl-2 levels were found in mitochondrial transplantation group. To explain this finding, it can be suggested that lower levels of Bak might decrease the necessity for increased in Bcl-2. Taken together, mitochondrial transplantation could be an interesting treatment candidate in sepsis challenge due to its anti-inflammatory and immune-enhancing properties [18]. Another study revealed the neuroprotective impacts of exogenous mitochondrial transplantation on modulating microglial polarization following sepsis. Mitochondrial transplantation mediated the microglial activation states, reduced M1 proinflammation phenotype, and increased M2 anti-inflammation phenotype. Additionally, it diminished cognitive impairment in sepsis survivors through microglial polarization from M1 to M2 phenotype. Altogether, mitochondrial transplantation modulated microglial polarization, suppressed the secretion of proinflammatory cytokine, and consequently improved long-term cognitive impairment following sepsis, indicating that it may be considered as a promising therapeutic strategy in sepsis-related brain dysfunction [19]. Zhang et al. showed that intravenous injection of extraneous mitochondria in CLP-induced sepsis improved survival rate of septic mice, which was associated with the reduction of systemic inflammation, bacterial burden, and organ damage. Extraneous mitochondrial replacement may be a beneficial strategy to diminish sepsis-related mortality [20].

## 6. Conclusion and Future Direction

Much of what are known about sepsis-induced mitochondrial damage and the effects of mitochondrial-based therapies in this context have been achieved from experimental studies. Given the presence of sparse data about sepsis-related mitochondrial dysfunction in septic patients, it would be interesting to evaluate mitochondria from vital organs such as the heart from septic patients over time. However, isolated mitochondria or tissue homogenates are required for evaluating mitochondrial function. As clinical studies are mainly limited to blood or skeletal muscle samples, to what extent data derived from blood cells can reflect whole body mitochondrial damage requires additional clarification [113]. Also, it is necessary to address the gaps and challenges in our knowledge about sepsis-induced mitochondrial damage and the impacts of mitochondrial-based therapies as well as mitochondrial transplantation on sepsis-associated organ dysfunction. One of these challenges is that experimental models of sepsis differ in many aspects from clinical studies. Most experimental studies have been modeled on animals that are often young without comorbidity, and of a single gender and similar genetic background far from the patient situation. So, there are limitations in translating the results of experimental studies to clinical setting. Also, experimental studies are commonly performed on short-term sepsis models (shorter than 24 hours), while in the clinical setting, most septic patients develop over several hours or days. Therefore, the reliability of experimental models of sepsis has been questioned [40].

Recovery of mitochondrial function in septic heart depends on a complex balance between prevailing levels of mitochondrial stress, autophagy signaling, biogenesis, and quality control processes, rather than a single factor. This is a complex issue, and the general mechanisms by which acute inflammatory conditions cause myocardial dysfunction and bioenergetics derangement need further evaluations. Importantly, when permanent mitochondrial damage occurs, reducing metabolic requirements and/or optimizing the residual ability of the cell to produce energy may counteract with dropping ATP level below the threshold that induces death pathways. This means that strategies that prevent or reverse mitochondrial damage and energetic failure in the cell may introduce a new treatment option for sepsis [52]. In order to develop mitochondrial-based strategies in clinical setting, further researches are needed for clarifying the timing and mechanisms of mitochondrial dysfunction in sepsis [59]. The association between amelioration of mitochondrial dysfunction and improvement of organ function has been shown in animal models of sepsis. Therefore, performing applicable clinical trials is essential to determine the role and timing of mitochondrial-based therapies in sepsis-related organ dysfunction in patients [14]. Researches for finding effective treatments for sepsis are still ongoing, and although the data from early studies are promising, there is still a long way to go. Given that removal of sepsis-related damaged mitochondria and production of normal mitochondria improve organ function [9], mitochondrial transplantation seems to be a promising therapeutic opportunity in sepsis. However, few studies have been performed about the positive effects of mitochondrial transplantation on organ damage in sepsis [18–20], and more studies are needed in this field.
